# Supplementary material for: Circulatory Rejuvenated EPCs Derived from PAOD Patients Treated by CD34+ Cells and Hyperbaric Oxygen Therapy Salvaged the Nude Mouse Limb against Critical Ischemia
Source: Int J Mol Sci. 2020 Oct 23;21(21):7887. doi: 10.3390/ijms21217887 (PMC7660611; doi:10.3390/ijms21217887)
Supplement: Supplementary file 1 [file ijms-21-07887-s001.pdf]

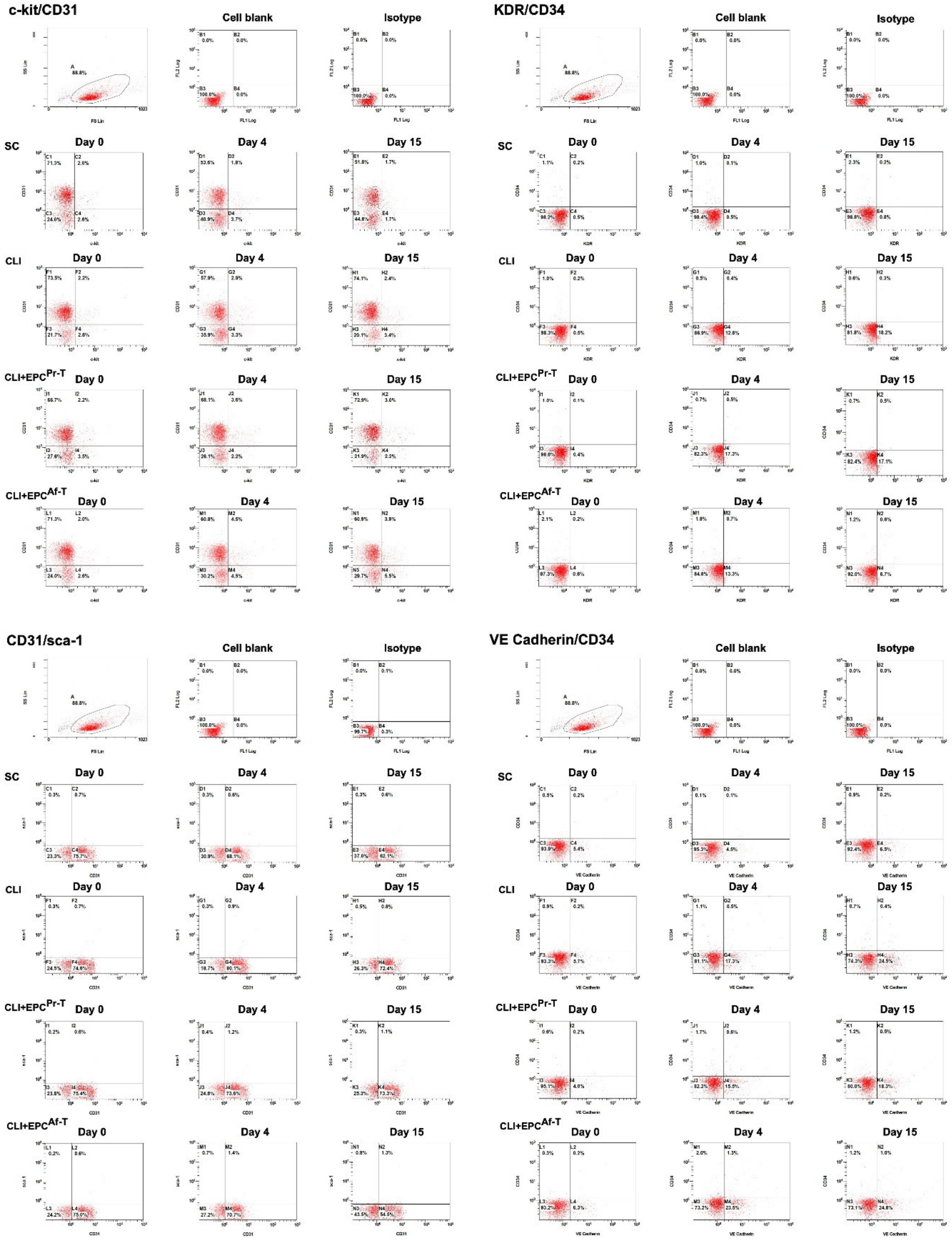

Supplementary Figure S1. Figures of flow cytometric analysis.for identification of circulating EPCs.
